# Hsa_circRNA_100146 Promotes Prostate Cancer Progression by Upregulating TRIP13 *via* Sponging miR-615-5p

**DOI:** 10.3389/fmolb.2021.693477

**Published:** 2021-07-07

**Authors:** Liang Zeng, Yi-min Liu, Ning Yang, Tao Zhang, Huang Xie

**Affiliations:** ^1^ Emergency Department, The Second Affiliated Hospital of University of South China, Hengyang, China; ^2^ Department of Anesthesiology, The Affiliated Nanhua Hospital, University of South China, Engyang, China; ^3^ Department of Urology, The Second Affiliated Hospital of University of South China, Hengyang, China

**Keywords:** CircRNA_100146, miR-615-5p, TRIP13, progression, prostate cancer

## Abstract

**Objective:** This study was conducted for investigating the functions of circular RNA circRNA_100146 (circRNA_100146) in the development of prostate cancer (PCa) and identifying the underlying mechanisms of the circRNA_100146/miR-615-5p/TRIP13 axis.

**Materials and Methods:** Under the support of RT-PCR, the expression of circRNA_100146 in PCa cells was examined. Cell Counting Kit-8 (CCK-8) assays and clone formation assays were applied to the assessment of cell proliferation. We then determined cell invasion and migration through transwell assays and wound healing assays. RNA pull-down assays and luciferase reporter assays were performed for the exploration of the regulatory effects of potential molecules on the expressions of the targeting genes. In addition, a nude mouse xenograft model was applied to demonstrate the oncogenic roles of circRNA_100146 in PCa.

**Results:** CircRNA_100146 expression was distinctly upregulated in PCa cells. Silencing of circRNA_100146 suppressed PCa cells’ invasion, migration, and proliferation. CircRNA_100146 sponged miR-615-5p to suppress its expressions, while miR-615-5p targeted the 3’-UTR of TRIP13 to repress the expression of TRIP13. In addition, we observed that knockdown of miR-615-5p reversed the suppression of circRNA_100146 silence on the proliferation and invasion of PCa cells. In addition, the tumor growth was also suppressed by silencing circRNA_100146 *in vivo*.

**Conclusion:** CircRNA_100146 is a tumor promoter in PCa, which promoted progression by mediating the miR-615-5p/TRIP13. CircRNA_100146 can be a potential candidate for targeted therapy of PCa.

## Introduction

Prostate cancer (PCa) is the second largest cause of human malignancy throughout the world (Wang et al., 2018). According to the epidemiological investigations, in the last twenty years, advanced countries exhibited deterioration in the incidence and mortality of PCa patients, followed by underdeveloped countries (Chang et al., 2014). Local therapies are useful for about one-third of patients with PCa who then develop metastatic diseases (Gonzalez et al., 2008). The PCa specimens with distant metastasis might result in androgen-independent PCa and hormone refractory PCa, which was considered to be one of the major causes of death in PCa patients (Wang et al., 2012; Wright et al., 2013). Although more and more achievements have been made on the improvements of the treatment efficiencies of PCa, a poor understanding of the mechanisms that drive the oncogenesis of the tumors has limited the development of better treatment outcome.

Circular RNAs (circRNAs) are closed-loop. They originate from the process of RNA transcription fragments connecting in an end-to-end way during transcription (Chen and Yang, 2015). Most circRNAs are conserved and expressed in a specimen- or cellular type–specific manner. More and more studies identify circRNAs as regulators in the expressions of various genes in diverse levels *via* interacting with DNAs, miRNAs, lncRNAs, or proteins to exhibit regulatory effects on various cellular physiological and pathological processes (Salzman, 2016; Kristensen et al., 2019). Recently, many types of tumors have shown a dysregulated expression of circRNAs (Meng et al., 2017; Yu et al., 2019). It has been proven that circRNAs are involved in the modulation of tumor-related genes by playing the role of protein-coding RNAs or competitive endogenous RNAs (ceRNAs) (Tay et al., 2014; Qi et al., 2015). However, the potential roles and molecular mechanisms of circRNAs remained largely unclear.

Thyroid hormone receptor interacting protein 13 (TRIP13) displays a functional regulatory effect on mitotic processes (Lu et al., 2019). In recent years, more and more studies reported the tumor-promotive roles of TRIP13 in many types of tumors, including PCa (Banerjee et al., 2014; Chen et al., 2020). However, the potential mechanisms involved in TRIP13 dysregulation remained largely unclear.

Hsa_circRNA_100146 (circRNA_100146) was newly identified to be a tumor-related circRNA. Its overexpression reportedly appears in some tumors like bladder cancer and lung cancer (Chen et al., 2019; Wang et al., 2020a). In addition, the oncogenic roles of circRNA_100146 were also demonstrated in the above tumors. However, in other types of tumors, rare research reported the expression and function of circRNA_100146. Herein, circRNA_100146 expression was found to be distinctly upregulated in PCa. Then, many functional assays were performed for exploring its biological function and the related mechanisms in PCa progression.

## Materials and Methods

### Cell Culture and Transfection

The American Type Culture Collection (ATCC, Manassas, VA, United States) was applied to the collection of normal myofibroblast stromal cell lines (WPMY1) and human PCa cell lines (LNCaP, VCaP, 22RV1, DU145, and PC-3). In an incubator with a humidified atmosphere and 5% CO_2_, cell culture was carried out in DMEM (Aiyan Teochnology, Pudong, Shanghai, China) containing 100 U/mL penicillin, 100 mg/ml streptomycin, 3 mM l-glutamine, and 10% fetal bovine serum at 37 °C.

Inhibitors, MiR-615-5p mimics, and their negative controls came from RiboBio. Specific siRNA oligonucleotides that targeted circRNA_100146 or TRIP13, and negative control (NC) siRNA came from Wuhe Biology (Fengxian, Shanghai, China). The transfected sequences of the above factors are shown in Supplementary Table S1. Based on the manufacturer’s protocols, Lipofectamine 3,000 (Invitrogen, Guangzhou, Guangdong, China) was used to conduct cell transfections. Western blot assays and RT-qPCR were used to achieve the detection of transfection efficiency.

### RNA Isolation and Quantitative Real-Time Polymerase Chain Reaction (qRT-PCR)

Cells with TRIzol (Invitrogen, Guangzhou, Guangdong, China) were used to extract total RNA. By the use of the miScript II RT Kit (Qiagen, Haidian, Beijing, China), cDNA synthesis was performed with 3 mg of total RNAs. Real-time PCR was carried out by the miScript SYBR Green PCR Kit (Qiagen) on the ABI 7,600 cycler (Applied Biosystems). GAPDH and U6 were used as endogenous controls. 2-ΔΔCt methods were used to calculate the relative expressions. Table 1 listed the sequence of the used primers.

**TABLE 1:** Primers used in this study for RT-PCR.

Names	Sequences (5′–3′)
circRNA_100146: F	GAG​CTC​AAC​CAG​TAT​AGT​GCC
circRNA_100146: R	ACA​TGA​TGA​TGT​TGC​CCC​CAA
miR-615-5p: F	GCA​TTT​AGC​AGC​GAG​ACA​A
miR-615-5p: R	AGC​GAC​ACG​TGC​GAA​TGT​TCT
TRIP13: F	ATC​CCA​TCT​CCT​CGA​TTA​TGT​GA
TRIP13: R	GGG​CTA​ACG​CTT​TAC​ACA​GGG
GAPDH: F	GCC​ACA​TCG​CTC​AGA​CAC​CAT
GAPDH: R	CCC​ATA​CGA​CTG​CAA​AGA​CCC
U6: F	TGC​GGG​TGC​TCG​CTT​CGG​CAG​C
U6: R	GTGCAGGGTCCGAGGT

### RNA Isolation and RNase R Treatments

TRIzol reagent was used to collect RNAs from 22RV1 and DU145 cells, which were then treated by DNase I (Ambion, Pudong, Shanghai, China) (37°C, half an hour, two times). The use of the Ribosomal Eukaryotic Kit (Qiagen, Chengdu, Sichuan, China) aimed to remove ribosomal RNAs. The purified RNAs were treated with RNase R (Jisai Biology, Xuhui, Shanghai, China), and then purified using TRIzol.

### Isolation of Nuclear and Cytoplasmic Fractions

NE-PER Nuclear and Cytoplasmic Extraction Reagents (Thermo Scientific, Guangdong, Shenzhen, China) were used to prepare cytoplasmic and nuclear fractions; qRT-PCR was used to test circRNA_100146 expression.

### Cell Counting Kit-8 (CCK8) Assays

The Cell Counting Kit 8 (CCK-8; Laifu Biology, Nanjing, Jiangsu, China) assays were applied to the measurement of PCa cells’ proliferation *in vitro*. In brief, 48 h after transfection, a 48-well plate was used to seed a total of 3 × 10^3^ cells and culture them at 37 °C. At indicated time points, the culture medium in each well was added by 15 μL CCK-8 (8 mg/ml). A microplate reader was applied for the detection of each well’s absorbance at 450 nm after 1 h of incubation at 37 °C.

### Clone Formation Assay

After 24 h of transfection, a 6-cm culture dish was used to seed PCa cells at a density of 300 cells/well. These cells were cultured until they could be seen by naked eyes. Subsequently, supernatants were removed, followed by 20 min of cell staining with crystal violet. A digital camera was used to capture images of the colonies.

### Transwell Assays

1.5 × 10^5^ 22RV1 and DU145 cells/well were applied for invasion assays. Cells that invaded were fixed 20 min with 100% methanol, followed by a staining process of 30 min with 0.5% crystal violet (Baomanbio, Xuhui, Shanghai, China). Finally, in five random fields of each filter, a microscope (IX71; Olympus) was used to count cells.

### Wound Healing Assays

The 6-well culture plates whose density was 1.0 × 10^6^ cells/well were used to implant cells. After cell fusing, a pipette tip was scraped on the cell monolayer, and cells were seeded by an FBS-free medium. Then, the inverted microscope (Olympus, Japan) was used to photograph PCa cell lines at 0 and 48 h of incubation.

### Western Blot Assay and Antibodies

RIPA lysate, as well as protein enzyme inhibitor cocktail, was applied to the extraction of the protein of 22RV1 and DU145 cells. Afterward, we loaded and separated them in 4–12% SDS-PAGE and transferred proteins in the gel to PVDF membranes (Sigma, Pudong, Shanghai, China). It was followed by the incubation process with specific antibodies and 5% milk. GAPDH was used as a control. Antibodies directed against TRIP13 (1:1,000, ab64964, Abcam, United States) and GAPDH (1:1,000, ab181602, Abcam, United States) were purchased from Guyan Technology (Pudong, Shanghai, China).

### Luciferase Reporter Assays

Synthesis of full-length circRNA_100146, TRIP13-3’UTR, and their corresponding mutant versions with mutant miR-615-5p binding sites was conducted. They were then cloned into the luciferase reporter vector psiCHECK-2. The 24-well plates were used to seed 22RV1 and DU145 cells (5 × 104), and Lipofectamine 3,000 was used to co-transfect them with corresponding plasmids and microRNA mimics. After incubation for two days, the cells were lysed, and a Dual-Luciferase Assay Kit (Promega, Pudong, Shanghai, China) was applied to the examination of their relative luciferase activity following the manufacturer’s protocol.

### Statistical Analysis

SPSS 19.0 software (IBM Corporation, Armonk, NY, United States) was applied to overall statistical analyses, and mean ± SD was used to express data. Statistical analyses were based on one-way ANOVA and Student’s t-test. Statistical significant differences were identified at *p*-value <0.05.

## Results

### CircRNA_100146 Expression Upregulated in PCa

**FIGURE 1:**
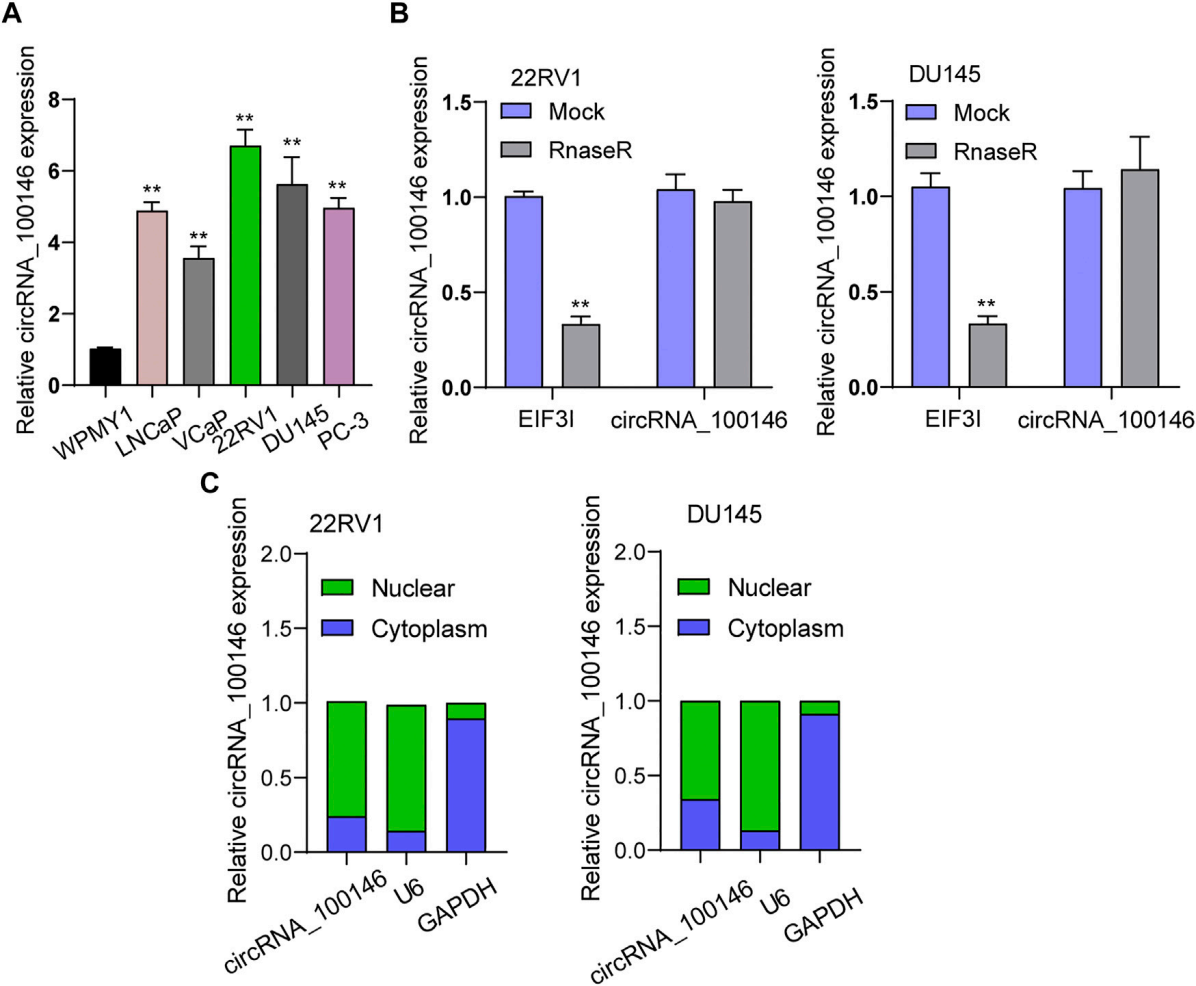
CircRNA_100146 expression was upregulated in PCa cells. **(A)** RT-PCR determined the expression of circRNA_100146 in five PCa cell lines and WPMY1 cells. **(B)** RNase R assays were carried out to verify the circular characteristics of circRNA_100146 in 22RV1 and DU145 cells. **(C)** RT-PCR was used to examine relative circRNA_100146 expression levels in nuclear and cytosolic fractions of 22RV1 and DU145 cells. **p< 0.01.

First, RT-PCR was applied to examine the levels of circRNA_100146 in PCa cells. As shown in Figure 1A, we observed distinctly upregulated circRNA_100146 expression in five PCa cells compared to WPMY1 cells. The RNase R experiment targeting 22RV1 and DU145 cells revealed resistance of circRNA_100146 to RNase digestion (Figure 1B). We also examined circRNA_100146 expressions in the nucleus and cytoplasm, which were confirmed by the results. Notably, circRNA_100146 in the cytoplasm presented higher expression abundance than that in the nucleus (Figure 1C).

### circRNA_100146 Silencing Inhibited the Proliferation and Metastasis of PCa Cells *In Vitro*

**FIGURE 2:**
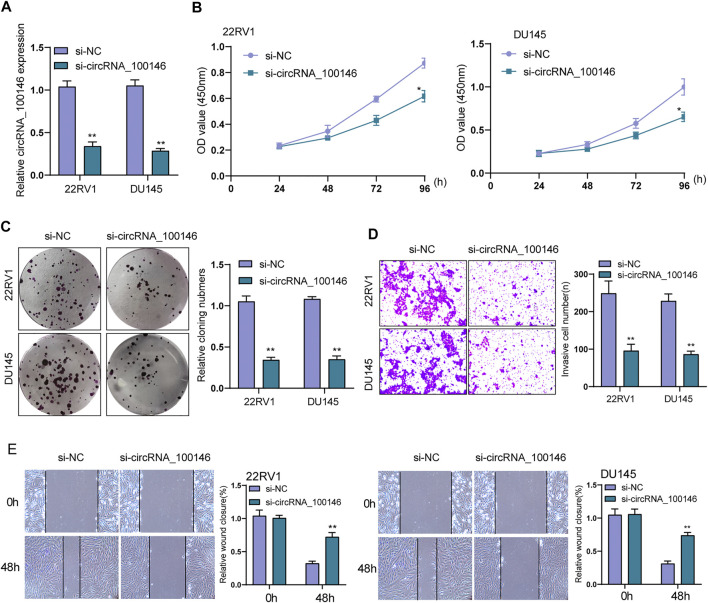
Effects of circRNA_100146 knockdown on the proliferation and metastasis of PCa cells. **(A)** The expression of circRNA_100146 in 22RV1 and DU145 cells transfected with si-NC or si-circRNA_100146. **(B)** CCK-8 assays for the proliferative determination in 22RV1 and DU145 cells after circRNA_100146 knockdown. **(C)** Cells with transfection of control siRNA or circRNA_100146 siRNAs were examined by colony formation assays. **(D)** Transwell assay indicated the invaded cell number in 22RV1 and DU145 cells transfected with si-circRNA_100146 and si-NC. **(E)** Representative images and quantitative analysis of scratch assays, and delayed closure was observed in 22RV1 and DU145 cells with circRNA_100146 knockdown. **p< 0.01, *p< 0.05.

We conducted loss-of-functional experiments *in vitro* the investigation of circRNA_100146’s biological influence on PCa cells. The expression level of circRNA_100146 in 22RV1 and DU145 cells was significantly impeded by siRNA targeting circRNA_100146 (si-circRNA_100146) transfection (Figure 2A). As found from CCK-8 and clone formation assays, si-circRNA_100146 obviously inhibited cell viability of 22RV1 and DU145 compared with si-NC (Figures 2B,C). Also, compared with si-NC, in both 22RV1 and DU145 cells, si-circRNA_100146 significantly inhibited both invasion ability (Figure 2D) and migration ability (*p* < 0.01) (Figure 2E). Our finding suggested circRNA_100146 as a tumor promoter in PCa progression.

### CircRNA_100146 Served as a Sponge of miR-615-5p

**FIGURE 3:**
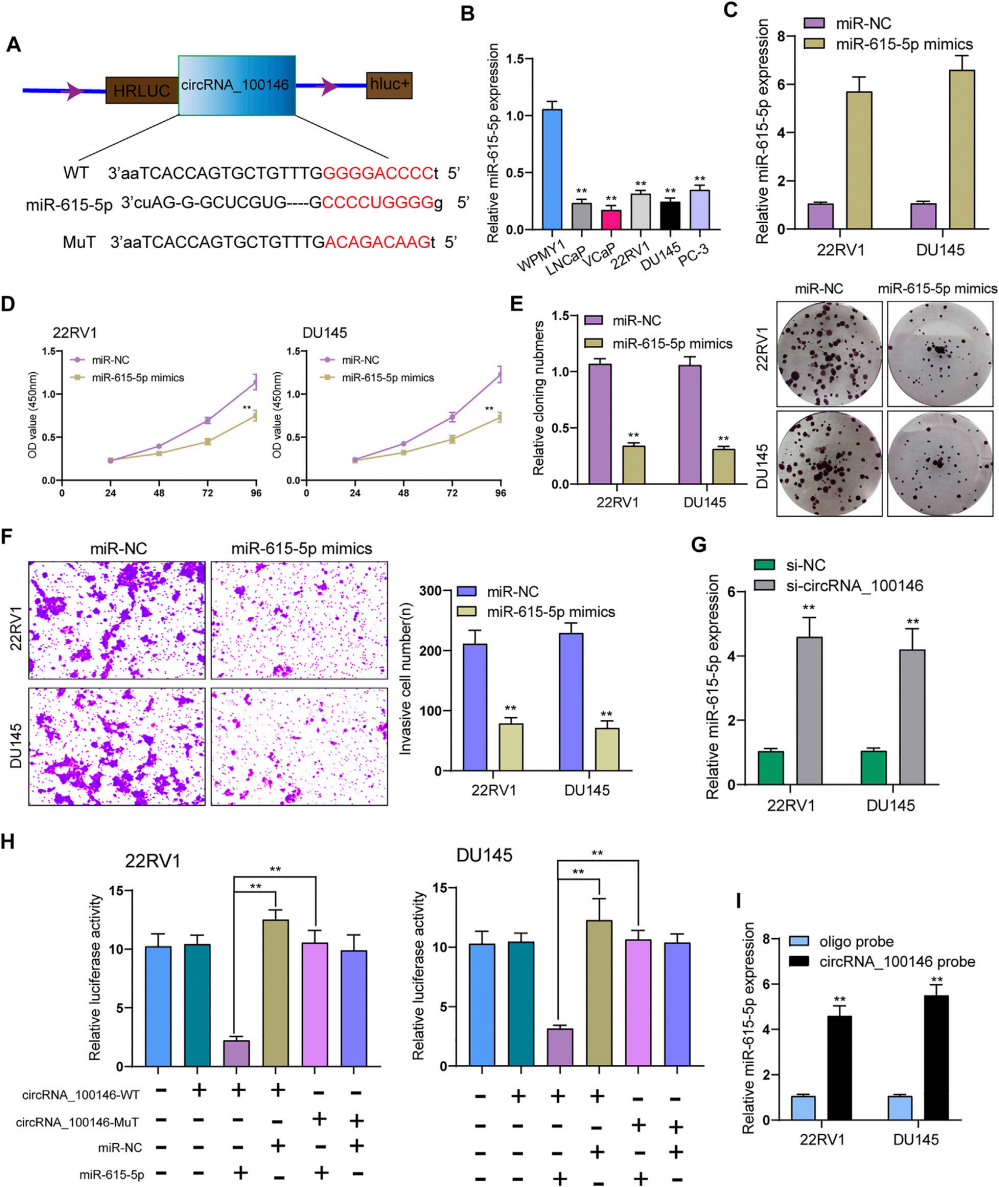
CircRNA_100146 interacts with miR-615-5p in PCa cells. **(A)** Bioinformatical predication of interaction between circRNA_100146 and miR-615-5p. **(B)** The levels of miR-615-5p in four PCa cells and WPMY1 cells by RT-PCR. **(C)** RT-PCR determined the expression of miR-615-5p in 22RV1 and DU145 cells transfected with miR-NC or miR-615-5p mimics. **(D)** CCK-8 assays were used to measure PCa cell proliferation after transfection. miR-615-5p overexpression significantly suppressed 22RV1 and DU145 cell proliferation. **(E)** The cell viability and proliferative activity of 22RV1 and DU145 cells were assessed by colony formation assay following transfection with miR-NC or miR-615-5p mimics, respectively. **(F)** Transwell assays were performed to determine the invasive abilities of si-circRNA_100146–transfected PCa cells. **(G)** miR-615-5p expression was decreased in 22RV1 and DU145 cells transfected with si-circRNA_100146 compared with those transfected with si-NC. **(H)** Relative luciferase activity after co-transfection of miR-615-5p mimics, miR-NC, circRNA_100146-Mut, and circRNA_100146-WT into 22RV1 and DU145 cells. **(I)** miR-615-5p was pulled down by circRNA_100146 in 22RV1 and DU145 cells. **p< 0.01.

ceRNA (competing endogenous RNA) network is an important mechanism by which circRNAs function (Karreth and Pandolfi, 2013). Whether circRNA_100146 regulated protein-coding gene by functioning as a ceRNA of miRNA was explored. It was predicted in Circular RNA Interactome database that MiR-615-5p served as a candidate target of circRNA_100146 (Figure 3A). Previously, miR-615-5p has been confirmed to be a suppressor of some tumors, including PCa. We also observed the decrease of miR-615-5p expression in PCa cells compared with WPMY1(Figure 3B). Functionally, PCa cells’ invasion and proliferation were hindered by (Figures 3C–F) overexpression of miR-615-5p. In addition, we also observed that the expression of miR-615-5p was obviously upregulated by knockdown of circRNA_100146 (Figure 3G). According to the results of luciferase assays, luciferase activity in WT was more strongly hindered by overexpression of miR-615-5p than Mut variants (Figure 3H). The data of RNA pull-down revealed that miR-615-5p was significantly pulled down compared to the control group (Figure 3I). Overall, miR-615-5p is a direct target downstream of circRNA_100146.

### CircRNA_100146 Regulates TRIP13 Expression *via* miR-615-5p

**FIGURE 4:**
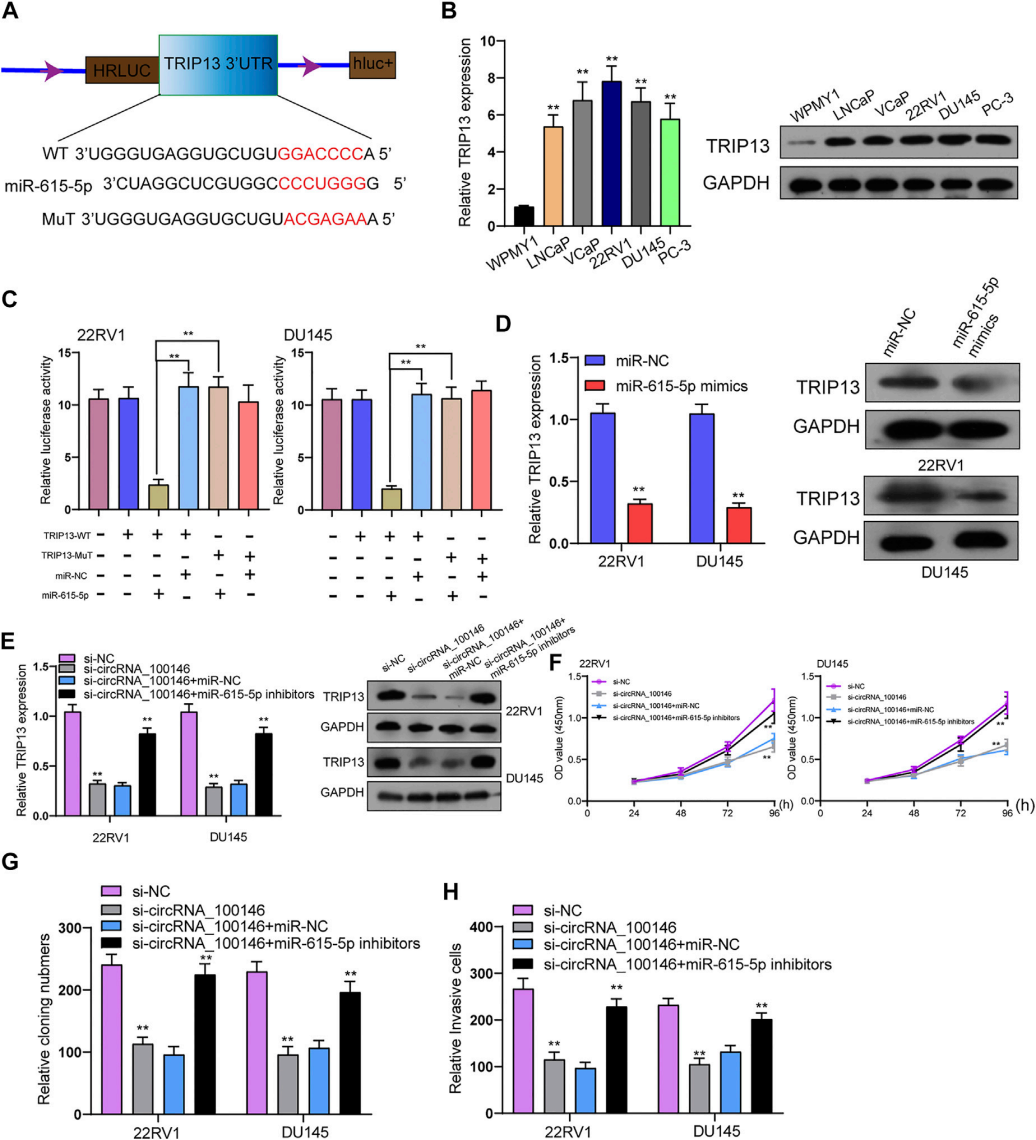
CircRNA_100146 decreased TRIP13 expression *via* sponging miR-615-5p. **(A)** Chematic illustration of the sequence of wild type of miR-615-5p 3’-UTR (WT) and mutant sequences on the complementary sites of miR-615-5p 3’-UTR with miR-615-5p (Mutant). **(B)** TRIP13 levels were increased in five PCa cells by RT-PCR and Western blot. **(C)** Relative luciferase activity after co-transfection of miR-615-5p mimics, miR-NC, TRIP13-Mut, and TRIP13-WT into 22RV1 and DU145 cells. **(D)** The transfection of miR-615-5p mimics resulted in the distinct suppression of TRIP13 expression in 22RV1 and DU145 cells. **(E)** RT-PCR and Western blot confirmed the expression of TRIP13 in 22RV1, and DU145 cells transfected with si-NC, si-circRNA_100146, si-circRNA_100146+miR-NC, or si-circRNA_100146+ miR-615-5p inhibitors. **(F–H)** The effects of circRNA_100146 knockdown on the proliferation and invasion were determined by CCK-8, colony formation assays, and transwell assays. **p< 0.01.

Further experiments were performed for exploring the functional executors of circRNA_100146 and miR-615-5p. As predicted by bioinformatics tools, the 3’-UTR of TRIP13 harbored the complementary binding sites with miR-615-5p (Figure 4A). TRIP13 has been confirmed to present high expression in PCa and accelerate PCa progression (Lu et al., 2019; Chen et al., 2020). In PCa cells, TRIP13 expression at both protein and mRNA levels was distinctly increased (Figure 4B). As found from the luciferase reporter assay, TRIP13 targeted the 3’-UTR of TRIP13 at molecular bound, which revealed the integration between TRIP13 and miR-615-5p (Figure 4C). Moreover, the transfection of miR-615-5p mimics was observed to result in the distinct suppression of TRIP13 expression in 22RV1 and DU145 cells (Figure 4D). Rescue experiments for exploring the association among circRNA_100146, miR-615-5p, and TRIP13 indicated that miR-615-5p inhibitors reversed the distinct suppression of circRNA_100146 knockdown on the expression of TRIP13 (Figure 4E). Moreover, functional investigations indicated that knockdown of circRNA_100146 suppressed the proliferation, migration, and invasion of 22RV1 and DU145 cells, which was reversed by the transfection of miR-615-5p inhibitors (Figures 4F–H). Overall, our findings suggested that circRNA_100146 may display its tumor-promotive roles *via* modulating miR-615-5p/TRIP13 axis.

### The Suppressor Effects of circRNA_100146 Silence Suppressed Tumor Growth *In Vivo*

**FIGURE 5:**
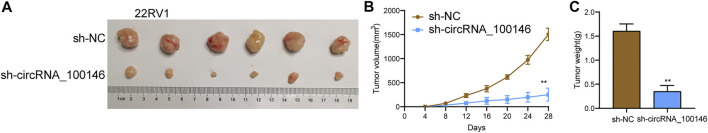
Oncogenic functions of circRNA_100146 knockdown on tumor growth. **(A)** Tumors from sh-NC or sh-circRNA_100146 group were shown. **(B,C)** Volume and weight of tumors obtained from sh-NC or sh-circRNA_100146 group were displayed. **p< 0.01.

For the exploration of the functions of circRNA_100146 on PCa, we introduced sh-NC– or sh-circRNA_100146–transfected 22RV1 cells into nude mice. Importantly, we observed that circRNA_100146 silence distinctly decreased tumor volume and weight when compared with the sh-NC group (Figures 5A–C).

## Discussion

Over the past years, increasing circRNAs have been reported to play a role in regulating PCa occurrence and development. For instance, high expression of circular RNA circANKS1B in PCa was reported, and its knockdown suppressed the proliferation and metastasis of PCa cells by upregulating TGF-α expression *via* sponging miR-152-3p (Tao et al., 2021). Circ_0006404 was shown to exhibit a higher level in PCa specimens than non-tumor specimens. Functional investigations revealed its overexpression displayed a positive role in the progression of tumor growth and metastasis *via* modulating miR-1299/CFL2 axis (Li et al., 2021). These findings highlighted the important effects of circRNAs on tumor developments. The previous literature has reported a substantial increase of circRNA_100146 in some tumors such as colorectal cancer, bladder cancer, and lung cancer (Chen et al., 2019; Wang et al., 2020a; Liu et al., 2021). Herein, in line with previous findings, circRNA_100146 expression was found to distinctly grow in PCa cells. Then, we performed a series of functional assays, finding that knockdown of circRNA_100146 suppressed PCa cells’ proliferation, migration, and invasion *in vitro* and *in vivo* experiments. Our results revealed circRNA_100146 as a tumor promoter in PCa progression. However, we just examined the expression of circRNA_100146 in PC cells. More PC specimens and non-tumor specimens from PCa patients were needed to further confirm whether circRNA_100146 was overexpressed in PCa.

Previous research works have proved that there is a widespread interaction network involving ceRNAs (Chan and Tay, 2018). Specifically, ncRNAs bind and titrate target RNA off their binding sites on protein-coding messengers for regulation (Wang et al., 2019). A close correlation exists between circRNAs functions and their subcellular localization (Esteller, 2011). Here, it was determined that circRNA_100146 was mainly localized to the cytoplasm in PCa cells, identifying its circRNA_100146 as a possible endogenous miRNA sponge. Previously, in bladder cancer and lung cancer cells, circRNA_100146 was also found to be mainly expressed in cytoplasm (Chen et al., 2019; Wang et al., 2020a). These findings suggested the location of circRNA_100146 in cytoplasm may be a common event in tumors. Then, we found circRNA_100146 may be a target of miR-615-5p. Previously, many studies have reported miR-615-5p served as a tumor promoter in several tumors (Guan et al., 2019; Godínez-Rubí and Ortuño-Sahagún, 2020). In PCa, miR-615-5p was shown to suppress the ability of proliferation and metastasis of tumor cells (Li et al., 2020). We also observed that the proliferation and metastasis of PCa cells were hindered by miR-615-5p overexpression. Thus, if circRNA_100146 plays the role of a ceRNA of miR-615-5p, it may display its oncogenic roles *via* sponging miR-615-5p. Importantly, luciferase reporter assays and RT-PCR confirmed miR-615-5p to be a target of circRNA_100146. The above results confirmed the interaction between circRNA_100146 and miR-615-5p contributed to lung tumorigenesis, given that circRNA_100146 exerts oncogenic function partly by sponging miR-615-5p in PCa cells.

As an AAA (ATPase family associated with various cellular activities) protein, TRIP13 belongs to a large AAA + protein superfamily of ring-shaped P-loop NTPases (Pfam: PF00004) (Vader, 2015). It plays a role in several cellular processes like checkpoint signaling, DNA break repair and recombination, and chromosome synapsis (Banerjee et al., 2014; Alfieri et al., 2018). In recent years, more and more studies confirmed that TRIP13 was frequently highly expressed in many types of tumors, including PCa (Zhang et al., 2019; Wang et al., 2020b; Chen et al., 2020). Functionally, several studies reported that TRIP13 overexpression promoted PCa cells’ proliferation and metastasis (Chen et al., 2020; Li et al., 2020). In this study, we found that TRIP13 may be a target of miR-615-5p, as further demonstrated by luciferase reporter assays. Thus, we wondered whether circRNA_100146 may increase the expression of TRIP13 by sponging miR-615-5p. Using rescue experiments, we observed that knockdown of miR-615-5p reversed the suppression of circRNA_100146 downregulation on the expression of TRIP13. In addition, by a series of functional assays, our group confirmed that knockdown of miR-615-5p reversed the inhibition of circRNA_100146 downregulation on PCa cells’ proliferation, migration, and invasion. Overall, our findings suggested circRNA_100146 promoted PCa progression *via* modulating miR-615-5p/TRIP13 axis.

## Conclusion

In summary, our results indicated distinctly increasing circRNA_100146 expressions in PCa. Furthermore, an inverse relationship was found between circRNA_100146 and miR-615-5p, and knockdown of circRNA_100146 exerted its tumor-suppressive effects at least in part through regulating miR-615-5p to modulate TRIP13 expression. Therefore, our study proved that circRNA_100146 could be a potential therapeutic target for the treatment of PCa.

## Data Availability

The raw data supporting the conclusions of this article will be made available by the authors, without undue reservation.
