# Supplementary material for: Hsa_circRNA_100146 Promotes Prostate Cancer Progression by Upregulating TRIP13 via Sponging miR-615-5p
Source: Front Mol Biosci. 2021 Jul 7;8:693477. doi: 10.3389/fmolb.2021.693477 (PMC8292639; doi:10.3389/fmolb.2021.693477)
Supplement: Supplementary file 1 [file Table1.DOCX]

**Table S1** Sequences of miRNA mimics, inhibitors, siRNAs of circRNA_100146, and their NC.

| Names | **Sequence** |
| --- | --- |
| Si-circRNA_100146 | GTATAGTGCCAAGGAAAGC |
| Si-NC | TTCTCCGAACGTGTCACGT |
| MiR-615-5p mimics | GGGGGTCCCCGGTGCTCGGATC |
| NC mimics | TACGTCAGGAACGTCAGTCCA |
| MiR-615-5p inhibitors | GATCCGAGCACCGGGGACCCCC |
| NC inhibitors | CCAGTTAAUAGUATTGTGT |
